# Supplementary material for: Smooth Interpolating Curves with Local Control and Monotone Alternating Curvature
Source: Comput Graph Forum. 2022 Oct 6;41(5):25–38. doi: 10.1111/cgf.14600 (PMC9827861; doi:10.1111/cgf.14600)
Supplement: Supplementary file 1 — Supplement Material [file CGF-41-25-s001.zip › Local-Smooth-Interpolating-MonoCurvature/extern/clothoids/submodules/Utils/ThirdParties/zlib-ng/zlib-ng-2.0.6.zip!/zlib-ng-2.0.6/test/GH-979/pigz-2.6.tar.gz!/pigz-2.6/pigz.pdf]

NAME

pigz, unpigz − compress or expand files

SYNOPSIS

pigz [**−cdfhikKlLmMnNqrRtz0..9,11**] [**-b** *blocksize*] [**-p** *threads*] [**-S** *suffix*] [*name ...*]
unpigz [**−cfhikKlLmMnNqrRtz**] [**-b** *blocksize*] [**-p** *threads*] [**-S** *suffix*] [*name ...*]

DESCRIPTION

Pigz compresses using threads to make use of multiple processors and cores. The input is broken up into 128 KB chunks with each compressed in parallel. The individual check value for each chunk is also calculated in parallel. The compressed data is written in order to the output, and a combined check value is calculated from the individual check values.

The compressed data format generated is in the gzip, zlib, or single-entry zip format using the deflate compression method. The compression produces partial raw deflate streams which are concatenated by a single write thread and wrapped with the appropriate header and trailer, where the trailer contains the combined check value.

Each partial raw deflate stream is terminated by an empty stored block (using the Z_SYNC_FLUSH option of zlib), in order to end that partial bit stream at a byte boundary. That allows the partial streams to be concatenated simply as sequences of bytes. This adds a very small four to five byte overhead to the output for each input chunk.

The default input block size is 128K, but can be changed with the **-b** option. The number of compress threads is set by default to the number of online processors, which can be changed using the **-p** option. Specifying **-p 1** avoids the use of threads entirely.

The input blocks, while compressed independently, have the last 32K of the previous block loaded as a preset dictionary to preserve the compression effectiveness of deflating in a single thread. This can be turned off using the **-i** or **--independent** option, so that the blocks can be decompressed independently for partial error recovery or for random access. This also inserts an extra empty block to flag independent blocks by prefacing each with the nine-byte sequence (in hex): 00 00 FF FF 00 00 00 FF FF.

Decompression can’t be parallelized, at least not without specially prepared deflate streams for that purpose. As a result, *pigz* uses a single thread (the main thread) for decompression, but will create three other threads for reading, writing, and check calculation, which can speed up decompression under some circumstances. Parallel decompression can be turned off by specifying one process (**-dp 1** or **-tp 1**).

All options on the command line are processed before any names are processed. If no names are provided on the command line, or if "-" is given as a name (but not after "--"), then the input is taken from stdin.

Compressed files can be restored to their original form using *pigz -d* or *unpigz.*

OPTIONS

-# --fast --best

Regulate the speed of compression using the specified digit #, where **−1** or **−−fast** indicates the fastest compression method (less compression) and **−9** or **−−best** indicates the slowest compression method (best compression). **-0** is no compression. **−11** gives a few percent better compression at a severe cost in execution time, using the zopfli algorithm by Jyrki Alakuijala. The default is **−6.**

-A --alias xxx

Use xxx as the name for any --zip entry from stdin (the default name is "-").

-b --blocksize mmm

Set compression block size to mmmK (default 128KiB).

-c --stdout --to-stdout
Write all processed output to stdout (won’t delete).

-C --comment ccc
Include the provided comment in the gzip header or zip central file header.

-d --decompress --uncompress
Decompress the compressed input.

-f --force
Force overwrite, compress .gz, links, and to terminal.

-h --help
Display a help screen and quit.

-H --huffman
Compress using the Huffman-only strategy.

-i --independent
Compress blocks independently for damage recovery.

-k --keep
Do not delete original file after processing.

-K --zip
Compress to PKWare zip (.zip) single entry format.

-l --list List the contents of the compressed input.

-L --license
Display the *pigz* license and quit.

-m --no-time
Do not store or restore the modification time. -Nm will store or restore the name, but not the modification time. Note that the order of the options is important.

-M --time
Store or restore the modification time. -nM will store or restore the modification time, but not the name. Note that the order of the options is important.

-n --no-name
Do not store or restore the file name or the modification time. This is the default when decompressing. When the file name is not restored from the header, the name of the compressed file with the suffix stripped is the name of the decompressed file. When the modification time is not restored from the header, the modification time of the compressed file is used (not the current time).

-N --name
Store or restore both the file name and the modification time. This is the default when compressing.

-p --processes n
Allow up to n processes (default is the number of online processors)

-q --quiet --silent
Print no messages, even on error.

-r --recursive
Process the contents of all subdirectories.

-R --rsyncable
Input-determined block locations for rsync.

-S --suffix .sss
Use suffix .sss instead of .gz (for compression).

-t --test
Test the integrity of the compressed input.

-U --rle
Compress using the run length encoding strategy.

-v --verbose
Provide more verbose output.

-V --version
Show the version of pigz. -vV also shows the zlib version.

-z --zlib
Compress to zlib (.zz) instead of gzip format.

-- All arguments after "--" are treated as file names (for names that start with "-")

These options are unique to the -11 compression level:

-F --first
Do iterations first, before block split (default is last).

-I, --iterations n
Number of iterations for optimization (default 15).

-J, --maxsplits n
Maximum number of split blocks (default 15).

-O --oneblock
Do not split into smaller blocks (default is block splitting).

COPYRIGHT NOTICE

This software is provided ’as-is’, without any express or implied warranty. In no event will the author be held liable for any damages arising from the use of this software.

Copyright (C) 2007-2021 Mark Adler <madler@alumni.caltech.edu>
